# Supplementary material for: Are carbon emissions trading and green financial instruments synergistic? -Comprehensive quantitative research based on content analysis
Source: PLoS One. 2024 Mar 7;19(3):e0298601. doi: 10.1371/journal.pone.0298601 (PMC10919601; doi:10.1371/journal.pone.0298601)
Supplement: S1 Data — (ZIP) [file pone.0298601.s001.zip › Data/地级市Z_F工作报告.zip!/║╙─╧/╫ñ┬φ╡Ω╩╨/└┤╫╘╣1⁄2╓┌║┼í╛┬φ┐╦╩2╛▌═°í┐_2015.docx]

各位代表：

现在我代表驻马店市人民政府，向大会报告工作，请予审议，并请各位政协委员和其他列席人士提出意见。

一、2014年工作回顾

过去的一年，面对极其复杂严峻的形势，我们在省委、省政府和市委的正确领导下，深入贯彻落实党的十八大、十八届三中、四中全会和习近平总书记系列重要讲话精神，紧紧围绕“推发展、促改革、保稳定、抓活动”四项重点工作，保持定力、集中精力、凝心聚力，维护了干部队伍的思想稳定、工作稳定和社会大局稳定，营造了浓厚的干事创业氛围和良好的发展环境，全市上下团结拼搏、务实重干，较好完成了驻马店市三届人大五次会议确定的各项任务。初步核算，全年完成生产总值1691.3亿元，增长8.4%；规模以上工业增加值509.2亿元，增长10.5%；固定资产投资1112.4亿元，增长19.7%；社会消费品零售总额612.3亿元，增长13%；财政一般公共预算收入85.7亿元，增长19.1%；城镇居民人均可支配收入21570元，农民人均纯收入8310元，分别增长9.7%和11.1%；节能减排完成省定目标；“四个驻马店”建设开局破题，科学发展的路径更加清晰，赶超发展的劲头更加充足，加快发展的动力更加强劲。特别是以下四组数据难能可贵：

——全市财政一般公共预算支出增长13.5%、金融机构存款余额增长16%、贷款余额增长24%、涉农贷款余额增长28.2%，四项指标增速全省第一；农业保险保费收入占全省的15.6%，总量全省第一。

——全市财政一般公共预算收入增长19.1%、商品房销售增长28.1%、小微企业贷款增长40.2%，三项指标增速全省第二；全市商品房销售面积736万平方米，总量全省第二。

——全市服务业增加值增长10.2%、社会消费品零售总额增长13%，两项指标增速全省第三；全市培育省级农业产业化集群10个，总量全省第三。

——全市固定资产投资增长19.7%、实际到位省外资金增长16.9%、完成进出口增长24.6%，三项指标增速全省第四。

过去一年，面对困难和挑战，我们迎难而上，重点做了以下工作：

(一)抓项目，着力实施投资拉动。坚持把项目建设作为稳增长、促转型的关键措施，深入开展项目建设推进年活动，认真落实周例会、月通报和领导分包等制度，先后两次开展项目观摩评比活动，推动了重点项目的顺利实施。全年分四批集中开工171个亿元以上重点项目，总投资853.7亿元，年度投资263.6亿元。全市257个重点项目完成投资602.4亿元，实现了重点项目投资突破500亿元的目标。其中，重点工业项目完成投资403亿元，交通和基础设施项目完成投资84亿元，服务业项目完成投资86亿元，分别较上年增加47亿元、43亿元和63亿元，银泰新能源汽车、海川电子玻璃等65个工业项目建成投产，金龙沃诚电动汽车、创新思念食品等61个业项目开工建设，京港澳高速改扩建等34个重点基础设施项目顺利推进。

(二)抓招商，着力实施开放带动。把开放招商作为“一举求多效”的重要举措，深入开展大招商活动，坚持“二分之一”工作法、月通报等制度，强化合同履约率、资金到位率、项目开工率督查，进一步提高了招商引资实效。全年签约亿元以上招商项目388个，合同总投资2242.8亿元，其中“中国农洽会”签约亿元以上重点项目75个，总投资641亿元。德威电缆、福汉兴公路港、天中古镇、中部汽贸物流园等一批重大项目成功签约。全年实际到位省外资金204亿元，增长16.9%；实际利用外资3.38亿美元，增长11.4%；完成进出口4亿美元，增长24.6%。

(三)抓工业，着力做大总量做优质量。受宏观经济的影响，去年驻马店市工业形势非常严峻， 2、8、9三个月全市工业用电量负增长，规模以上工业增加值增速下滑、低位徘徊，其中4月份仅增长5.6%，为建市以来最低。面对这一形势，我们深入开展企业服务和减轻企业负担活动，切实加强对工业运行的分析和监测，着力扩大增长点、转化拖累点、抓好关键点、抢占制高点，促进了工业经济的稳步回升。全年工业用电量增长8.5%，增速居全省第五位、黄淮四市第一位；全市规模以上工业主营业务收入增长14.2%，实现利润增长9.5%。一批重点企业快速发展，昊华骏化主营业务收入突破百亿元，中集华骏、蓝天集团分别达到65亿元、51.5亿元。科技创新成效明显，全市高新技术企业达到13家、创新型企业68家，建立博士后工作站2个、重点实验室5个，获省科技进步奖6项，“中科院河南产业创新与育成中心驻马店分中心”挂牌运行。加强产业集聚区基础设施和配套服务设施建设，大力培育主导产业和产业集群，深入开展晋档升级活动，全市产业集聚区完成固定资产投资786亿元，规模以上工业主营业务收入1710亿元，分别占全市的70.7%和77.6%；确山县产业集聚区被评为全省“十快”产业集聚区，遂平、汝南、确山、上蔡4个产业集聚区被认定为一星级产业集聚区，全市一星级产业集聚区达到7个。

(四)抓城建，着力推进新型城镇化。认真落实《科学推进新型城镇化实施意见》，统筹推进中心城区、县城和重点镇建设，全市城镇化率达到37.5%，较上年提高1.5个百分点。城乡一体化示范区总体规划经市人大常委会审议通过，各项专业规划和重点区域规划正在编制。中心城区七大片区、十项城建重点工程有序推进，练江河片区河道治理加快实施，小清河水系治理通过立项评审；中原大道北段、创业大道西段、铜山大道北段等建成通车，纬十路、文明大道南段、置地大道东段、开源大道西段等开工建设，市体育中心等项目加快推进；市第一污水处理厂完成升级改造，第二、第三污水处理厂建成投运，中心城区日污水处理能力达到22.5万吨；城市管网等基础设施不断完善，新建改造供水、热力、燃气管网40多公里；把“香樟”确定为市树、“紫薇”确定为市花，明确了中心城区绿化主导品种，新增绿化面积96公顷。中心城区完成基础设施投资61亿元，185平方公里的大框架初步形成，建成区面积达到80平方公里。理顺了市区管理体制，开展了市容环境卫生集中整治活动。加强中心城区土地、规划和项目管理，成立了高规格的市土委会、规委会和项目评审委员会，完善了议事规则，规范了土地供应、规划审批和项目评审，城市建设逐步走上了规范化、法治化轨道。持续推进“双违”集中整治，依法拆除54处违法建筑，整改172处；明确了违法建筑属地管理责任，实行严厉的惩罚措施，遏制了“双违”建设蔓延势头。着力破解征地拆迁难题，制定出台了统一规范的中心城区征地拆迁安置政策，启动了重点项目集中征地拆迁工作。各县基本完成了县城总规编制工作，多方筹资加强基础设施建设，实施了一批城市综合体、片区开发项目，县城建设全面提速，发展亮点越来越多，城市功能逐步完善，管理水平不断提高，县城面貌发生了很大变化。按照突出重点、示范引领的原则，启动了30个美丽乡镇示范创建工作。

(五)抓三农，着力统筹城乡一体化发展。大力实施高标准粮田“百千万”工程，新建高标准粮田131万亩，全年粮食总产144.4亿斤，增长1.9%，实现了“十一连增”。积极推进农业产业化集群和新型农业经营主体培育工作，全市农业产业化集群发展到43个，其中省级农业产业化集群10个；全市新型农业经营主体15600多家，其中农民专业合作社10633家、家庭农场4445家，总量均居全省第一；土地流转面积249.6万亩，占家庭承包面积的24%。改善农村人居环境工作扎实推进，434个整治村环境明显改善。都市生态农业发展工程启动实施，创建国家级休闲农业与乡村旅游示范县1个、示范点3个、中国美丽田园1个。现代畜牧业持续发展，新建标准化规模养殖场105个，全市肉、奶总产量分别增长3.65%和1.5%，畜禽饲养量继续保持全省领先位次。完成造林16.9万亩，新改建农村公路780公里，解决了68.5万农村居民安全饮水和12.7万贫困人口脱贫问题。

(六)抓三产，着力培育新的经济增长点。坚持把发展服务业作为弥补短板和扩大消费的重点，召开了全市服务业发展大会，出台了《加快服务业发展的意见》，促进了服务业的快速发展。全市商务中心区和特色商业区建设全面启动，累计完成投资70亿元，市商务中心区重点项目加快实施，平舆商务中心区被评为全省“十快”商务中心区；房地产业健康发展，房屋施工、商品房销售面积分别增长19%和28.1%；现代物流业快速发展，市公共保税中心、恒兴物流等一批重点项目顺利实施；电子商务发展势头迅猛，筹建了市电子商务产业园，成立了全省首家跨境电子商务学院，阿里巴巴·驻马店产业带投入运营，已入驻企业200多家；旅游产业持续发展，大嵖岈旅游区、千年岭国家农耕文化公园、象河谷休闲度假区等重点项目加快推进，老乐山景区成功创建国家4A级景区，嵖岈山景区通过国家5A级景区景观质量评审，全市实现旅游业综合收入106.5亿元，增长25.6%。

(七)抓改革，着力破解发展难题。坚持用改革办法解决发展难题，发展的内生动力和市场活力不断增强。基本完成了市、县两级政府机构改革；大力推进行政审批制度改革，取消行政审批事项137项，减少近50%，中心城区开通了网上审批业务；工商注册便利化改革成效明显，全市新登记企业6177家，增长31%，激发了创业热潮。持续深化国有企业改革，县级公立医院综合改革全面实施。加大金融改革力度，平舆县、正阳县、泌阳县村镇银行组建工作已经银监会批准，驿城区、西平县农联社达到组建农商行条件。农村集体土地所有权确权登记基本结束，土地承包经营权确权登记工作顺利实施；文化、财税、户籍、交通执法等领域的改革稳步推进。

(八)抓民生，着力增进人民福祉。进一步加大民生投入力度，全年完成民生支出280亿元，占财政一般公共预算支出的80%。十项重点民生工程全面完成，中心城区六件惠民实事完成年度计划。持续推进学前教育三年行动计划，学前教育毛入园率达76%；优化基础教育资源配置，城镇义务教育教学水平不断提升，农村贫困地区义务教育办学条件持续改善。职教园区建设完成投资12亿元，驻马店高级技工学校、汝南幼儿师范搬迁入驻，农校、卫校建设进展顺利。深入实施卫生事业“三年九大攻坚计划”，加强乡镇卫生院服务能力和村卫生室标准化建设，新农合和城镇居民医保财政补助标准进一步提高，大病保险实现了省级统筹、即时结报，重大传染病防治工作不断加强。“单独两孩”生育政策顺利实施。文化事业取得新成效，驻马店日报报业集团挂牌成立，大型廉政历史剧《陈藩》获河南省第13届戏剧大赛文华大奖。残疾人事业持续发展，成功创建了“全国残疾人文化体育示范市”。全年城镇新增就业7.5万人，失业人员再就业3.4万人，新增农村劳动力转移就业7.5万人，城乡居民社会养老保险实现全覆盖，企业退休人员养老金、城乡低保标准进一步提高，新农合参合率达98.5%。全市新开工保障性住房3.7万套，基本建成2.2万套。在全省率先实现与全国34个城市“公交一卡通”，被确定为创建“省公交优先示范市”；大力倡导绿色低碳出行，中心城区公共自行车服务系统建成投用。深入实施“蓝天工程”、“碧水工程”、“乡村清洁工程”，全市完成81家燃煤小锅炉拆改任务，火电、钢铁、水泥行业脱硫脱硝全部完成，市区露天烧烤集中整治成效明显，淘汰黄标车及老旧车辆9364辆，超额完成省定任务；全市空气质量优良天数247天，超省定目标28天。加强和创新社会治理，积极开展信访突出问题集中整治活动，组织万名干部下基层，化解了一大批矛盾纠纷和信访积案；切实加强安全生产、食品药品安全和社会治安综合治理，严厉打击各类刑事犯罪，现行命案破案率连续五年实现100%，打击犯罪综合效能连续四年全省第一，人民群众的安全感、满意度持续提升，社会大局和谐稳定。

(九)抓活动，着力提升施政水平。深入开展党的群众路线教育实践活动和政府系统“转作风、提效能、破难题、惠民生”主题教育活动，加强学习教育，深入查摆问题，强化整改落实，政府作风建设和工作水平明显提升。认真落实中央八项规定和省、市若干意见，着力解决“四风”突出问题；严格执行国务院“约法三章”，政府性楼堂馆所、机关事业单位人员编制、“三公”经费得到有效控制。以制度建设规范行政行为，修订完善了市政府工作规则、常务会议议事规则，制定出台了重大行政决策听取意见、重点工作考评奖惩以及征地拆迁、群众安置、规划建设、项目审批等工作制度，集中力量破解了一批发展中的重大难题，形成了推动工作的良性机制，促进了政府工作的科学化、规范化、制度化。

一年来，我们自觉接受人大、政协监督和社会监督，制定了依法接受人大及其常委会监督和自觉接受政协民主监督的意见，认真办理人大代表建议和政协提案，支持民主党派、工商联和无党派人士参政议政。切实加强民主法制工作，大力推进依法行政和服务型执法，健全权力运行制约监督体系，强化行政监察和审计监督，积极推进党风廉政建设和反腐败斗争，营造了风清气正的发展环境。深入开展精神文明创建活动，全市有7人荣登“中国好人榜”，入选数量全省第二，驻马店市和平舆县被评为创建全国文明城市提名城市；深入开展国防教育，高度重视驻驻部队、国防后备力量和人民防空建设，持续开展双拥工作，推动了军民融合深度发展。民族宗教、统计、气象、侨务、史志、档案、红十字、妇女儿童、对口援疆等工作都取得了新的成绩。

各位代表!在经济下行压力大、困难挑战多的情况下，取得这些成绩来之不易。这是省委、省政府正确领导和关心支持的结果，是驻马店市委把握大局、科学运筹的结果，是市人大、市政协大力支持的结果，更是全市干部群众团结拼搏的结果。在此，我代表市政府向广大工人、农民、知识分子、干部，向驻驻部队指战员、武警官兵、公安民警，向所有关心支持政府工作的各位人大代表、政协委员、离退休干部、民主党派、工商联和无党派人士，表示崇高的敬意和衷心的感谢!

我们也清醒地认识到，当前驻马店市经济社会发展、政府自身建设方面还存在一些突出问题。一是经济下行压力较大，全市规模以上工业增加值、固定资产投资、一般公共预算收入等多项指标增速回落，尤其是生产总值增速低于预期，主要是去年我市规模以上工业增加值增幅回落明显，对经济增长造成了较大影响。二是经济总量小，发展质量不高，现代服务业和战略性新兴产业发展滞后，科技创新能力弱，工业化、城镇化水平低，引领经济增长的动力不足，加快发展、赶超发展任务艰巨。三是教育、卫生、文化等社会事业欠账较多，公共服务水平低，食品药品安全、安全生产等领域还存在不少薄弱环节，非法集资、征地拆迁、生态环境、城市管理、涉法信访等一些涉及群众切身利益问题尚未得到很好解决。四是政府职能转变、简政放权还不到位，行政管理的规范化、信息化水平还不适应新常态的要求，发展环境仍不宽松，一些地方、部门和工作人员作风不实、服务不优，为官不为、不敢担当以及“慵、懒、散”现象依然存在，一些领域仍然存在腐败现象。对此，我们一定高度重视、认真解决。二、2015年政府工作的主要任务

今年是全面完成“十二五”规划的收官之年，也是全面深化改革的关键之年。综合判断，当前世界经济正处于深度调整期，复苏动力不足、不确定因素增多，但经济全球化深入推进、产业跨国转移总体趋势没有改变；我国经济发展进入新常态，增速换挡、结构调整、动力转换压力增大，但仍处于可以大有作为的重要战略机遇期，总体向好的基本面没有改变；驻马店市与全国、全省一样，新常态下投资驱动力有所减弱，稳增长调结构难度加大，资源环境约束加剧，各类潜在风险累积叠加，但“四化”同步发展蕴藏的巨大需求潜力不断释放，我市要素资源、区位交通优势更加凸显，载体平台、重大项目等对发展的支撑能力不断增强，全市上下建设“四个驻马店”的信念坚定、热情高涨，蓄势崛起的基本态势没有改变。只要我们抢抓机遇、顺势而为，趋利避害、乘势而上，就一定能够牢牢把握发展的主动权，推动全市经济社会发展再上新台阶、实现新突破。

今年政府工作的总体要求是：全面贯彻党的十八大及十八届三中、四中全会和习近平总书记系列重要讲话精神，以邓小平理论、“三个代表”重要思想、科学发展观为指导，围绕“四个全面”战略部署，主动适应经济发展新常态，坚持调中求进、改中激活、转中促好、变中取胜，以提高经济发展质量和效益为中心，着力推动科学发展、着力调优经济结构、着力实施项目带动、着力推动开放招商、着力全面深化改革、着力增创发展优势、着力保障改善民生，促进经济持续健康较快发展和社会和谐稳定，推动富强、文明、平安、美丽驻马店建设迈出新步伐。

今年经济社会发展的主要预期目标是：生产总值增长8%以上，规模以上工业增加值增长11%，全社会固定资产投资增长17%，一般公共预算收入增长11%，社会消费品零售总额增长12%，城乡居民收入增长9%以上，城镇新增就业4.8万人，居民消费价格涨幅控制在3%以内，城镇化率提高1.5个百分点以上，节能减排完成省定目标，发展方式转变取得新进展。

实现上述目标，做好今年的政府工作，必须主动适应新常态，既要清醒认识新常态下增速换挡的必然性，把握经济规律，积极应对挑战；又要清醒认识新常态下的新机遇，进一步转变执政理念和发展方式，努力在新常态下取得新发展。必须千方百计做大经济总量，坚持把发展作为第一要务，把做大做强工业经济作为重中之重，推动产业优化升级，培育壮大优势产业和骨干企业，持续推进产业集聚、人口集中、土地集约，实现“三化”协调、“四化”同步。必须坚持在发展中优化结构，大力发展二三产业，推动产业结构优化升级。突出优化经济增量，特别是在招商引资和项目建设中，更加注重质量效益，更加注重节能降耗，更加注重新产业、新业态的培育，实现经济良性发展。必须坚持投资拉动和创新驱动并举，一方面坚定不移地推进开放招商和项目建设，努力扩大投资规模，进一步夯实发展基础；另一方面加强观念创新、科技创新、产品创新、业态创新和商业模式创新，培育新的经济增长点，推动全市经济持续健康发展。重点抓好九个方面的工作：

(一)大力实施开放招商，积极承接产业转移。深入实施开放招商战略，积极承接产业转移，不断提升招商水平，力争全年到位省外资金增长8%，实际利用境外资金增长8%。持续开展大招商活动。坚持把招商引资作为重中之重，认真落实“二分之一”工作法，组织强有力班子和精干队伍，瞄准重点区域和重点企业，围绕龙头企业招大引强，围绕产业链延伸招专引精，围绕发展方向招新引先，围绕资源优势招特引优，有针对性地开展招商活动，各县区每季度要组织开展一次专业招商活动。对一些重大招商项目，主要领导亲自挂帅，组成专业团队开展一对一、点对点洽谈对接，做到常年抓、不断线。继续组织筹办好中国农加工洽谈会，力求取得更大成效。切实提高招商实效。依托我市优势产业和现有骨干企业，不断转变招商方式，强化领导招商、以商招商、落地招商、规划招商，大力开展专项产业招商活动，集中引进一批全产业链、集群式的重大项目。对去年以来特别是中国农加工洽谈会上签约的项目，加强洽谈对接，搞好跟踪问效，确保项目尽早落地建设。强化招商基础工作，加强招商队伍建设，科学编制招商图谱，切实提高招商引资的实效。认真落实招商引资考核奖惩制度，强化督导检查，不断提高项目的合同履约率、资金到位率和项目开工率。着力构建大开放格局。积极融入“一带一路”战略，加强与郑州航空港经济综合试验区的对接，大力发展外向型经济，打造内陆开放新高地。按照非禁即入原则，推进全方位宽领域多层次开放，有针对性地筛选包装一批重大项目，搞好对外发布和推介，吸引社会资本和境外投资开发建设。不断优化开放环境。适应开放招商新形势，切实加强软硬环境建设，牢固树立“重商、亲商、安商、富商”的理念，实现由靠优惠政策招商向靠优化服务环境招商的转变。加强开放招商载体平台建设，抓好驻马店保税中心“两仓”建设和验收，为企业进出口提供便利条件。深入开展优化环境专项治理，打造良好的开放招商环境。

(二)强力推进项目建设，不断增强发展后劲。坚持把项目建设作为拉动投资、加快发展的重要抓手，持续开展项目建设推进年活动，进一步加大项目建设力度，确保全市固定资产投资突破1300亿元、产业集聚区固定资产投资突破900亿元。切实抓好重点项目建设。对今年全市初步安排的总投资2089亿元、年度投资648亿元的264个亿元以上重点项目，强化协调服务，加快建设进度，全面完成年度投资计划，确保重点项目投资占固定资产投资的70%以上。突出抓好工业项目建设。组织实施好总投资1080亿元、年度投资399亿元的194个重点工业项目，对德威电缆、平煤蓝天原料替代改造、华中正大和正大饲料搬迁等计划开工项目，积极落实开工条件，确保尽早开工建设；对天方药业新型原料药、恒丰车轮、闽商食品工业园等在建项目，加快建设进度，确保完成投资计划；对昊华骏化原料路线改造、中多铝业、十三香现代化生产等计划竣工项目，倒排工期、加快进度，促其按期竣工投产，确保全市工业项目投资占重点项目投资的70%以上。积极争取上级项目。重点围绕农业基础设施、保障性住房、交通、城市管网、节能环保、生态建设、结构调整、社会事业等领域，深入研究国家产业政策和投资方向，积极谋划包装项目，加大前期工作力度，争取周驻南和息邢高速、安信快速通道、南驻阜铁路等一批重大项目纳入国家、省“十三五”规划。鼓励推动大众创业。充分发挥政府引导作用，完善鼓励大众创业扶持政策，设立创业基金、搭建创业平台、健全服务体系，营造有利于大众创业的体制机制和政策环境。放宽市场准入，消除政策壁垒，降低创业门槛，完善金融服务，激发市场活力，支持社会资金参与基础设施建设、现代农业、服务业和社会事业发展。加快项目实施进度。积极推行模拟审批、全程代理和网上审批，加快项目审批进度。切实做好征地拆迁工作，科学安排、交叉推进、缩短周期，为项目落地创造条件。认真落实重点项目领导分包、周督导、月通报、季检查、半年总结、年终考评制度，按照“一线工作法”，通过现场会、早餐会等方式，加大问题协调解决力度，提高工作效率，为项目实施创造良好环境。解忧杂货店

(三)加快新型工业化，做大做强工业经济。大力推进工业强市战略，深入实施骨干企业、产业集群、战略性新兴产业、科技创新、上市企业培育“三年行动计划”，加快新型工业化发展。培育壮大骨干企业。认真实施“30强、30高、30新”培育工程，支持天方药业、后羿制药等企业创新发展，抢占行业制高点；支持昊华骏化等企业改造升级、延长链条，打造全省重要的煤化工基地；支持中集华骏、大力天骏加快发展，打造在全省有影响的汽车及零部件生产基地；支持徐福记、联顺食品等企业做大做强，打造全省重要的食品工业基地。力争昊化骏化主营业务收入超120亿元，中集华骏、蓝天集团超70亿元，一加一面业等30家企业超10亿元。大力发展战略性新兴产业。继续强化政策扶持，积极实施高端装备、电子信息、新能源、新材料、生物医药等重大项目和关联性配套项目建设，争取把电子玻璃、新能源汽车等新兴产业培育成主导产业。大力开展“十百千科技型企业”培育工程，力争培育高新技术后备企业8家，认定高新技术企业7家以上、创新型企业40家。强化企业主体作用，支持企业研发平台建设，力争培育3个省级、15个市级工程技术研发中心，承接实施一批国家和省级重大科研项目。力争全市新兴产业主营业务收入增长30%以上。进一步强化企业服务。重点围绕企业经营运行和扩大投资搞好服务，认真落实减轻企业负担的政策措施，推动企业服务常态化、制度化。对有竞争力、有销路的企业，加大扶持力度，支持企业开足马力生产；对生产经营困难企业，综合运用延伸链条、技术改造、淘汰落后产能等手段，帮助企业摆脱困境。对重点工业企业实行动态管理，从县区筛选一批具有发展潜力的企业纳入市重点企业管理。切实加强经济运行监测分析，搞好煤电油气运协调保障，确保工业经济平稳运行。加快产业集聚区发展。继续加强基础设施和配套设施建设，不断完善服务功能，提高承载能力和服务能力。进一步理顺体制机制，合理调整行政区划，年底前基本完成与区镇的管理套合。深入开展晋档升级活动，确保全市产业集聚区固定资产投资增长20%，规模以上工业增加值增长13%，全市7个产业集聚区实现主营业务收入超百亿元，市产业集聚区达到二星级，市装备产业集聚区和西平、正阳、市经济技术产业集聚区达到一星级。

(四)统筹城乡发展，科学推进新型城镇化。认真贯彻中央和省委、省政府决策部署，出台实施新型城镇化规划和三年行动计划，科学推进新型城镇化发展。不断提高城镇综合承载能力。持续加快中心城区建设，着力实施9大片区等100个重点城建项目，突出抓好小清河、练江河蓄水工程和片区综合开发，完成市区纬十路、置地大道东段、文明大道南段、练江大道西段和市体育中心建设任务；开工建设京港澳高速驻马店北出口，改造开源大道出口；实施市区铜山大道向南延伸、汝河大道向西延伸、乐山大道向北延伸和文明大道北段改造工程，打通市区天顺路、靖宇路、纬一路、纬二路等断头路；加快城市园林绿化、供排水、供气供热、防洪防涝等市政建设；启动实施驻马店高铁汽车客运枢纽站建设；以火车站片区综合改造为重点，完成中心城区37个棚户区改造项目，力争基础设施投资完成60亿元以上。大力实施县城扩容提质工程，加快基础设施和公共服务设施建设，每个县城基础设施投资完成10亿元以上。加强重点镇规划和建设，发挥30个重点镇的示范效应，着力打造一批功能完善、特色鲜明的新型城镇，争创一批国家园林县城和省级园林乡镇。提升城市管理水平。健全规划体系，积极推进“多规合一”，更好地发挥规划对城市建设的引领作用。深入开展“双违”整治活动，坚决遏制和严厉打击违规违法建设行为。加强建筑市场管理，明确主体责任，强化源头控制，实行黑名单制度，从根本上解决建筑市场的混乱局面。完善城市管理长效机制，划小网格、重心下移，下大力气治理城市污染、交通拥堵以及乱停乱放、乱贴乱画、乱摆摊设点、乱上路营运等城市“乱象”，让出行更方便、环境更优美。大力开展文明城市创建工作，加强文明教育，提升市民素质，树立城市形象。加快城乡一体化示范区建设。突出细化规划、拉大框架、融资开路、片区突破工作重点，尽快完成示范区专项规划编制工作；以市区开源大道、雪松大道向西延伸为重点加强基础设施建设；加快市商务中心区建设，启动高铁商务西区、都市农业示范片区和休闲旅游养生片区开发建设；注重体制机制创新，发挥统筹城乡发展的示范引领作用。有序推进农村人口转移。坚持“一基本两牵动三保障”，深化户籍制度改革，积极跟进教育、医疗、就业、社保等配套改革，优先解决已进城就业定居农民工落户问题，将城中村、产业集聚区内村庄居民成建制转为城镇人口。完善农民承包地流转、农村宅基地退出、农村居民集体财产权益保障等机制，切实保障进城农民的原有权益，消除农民进城的后顾之忧。

(五)持续加强三农工作，夯实现代农业发展基础。按照稳粮增收、提质增效、创新驱动总要求，加快发展方式转变，推进现代农业发展，努力实现农业强、农民富、农村美。稳定提高粮食综合生产能力。开展永久基本农田划定工作，实施耕地质量保护和提升行动，加强新技术、新品种、新农机推广应用，集中连片建设高标准粮田100万亩，确保粮食综合生产能力稳定在130亿斤以上。大力推进农业产业化。重点抓好夏南牛、黑猪、花生、花菇、芝麻五大特色农业产业化，加强标准化种植、养殖和加工基地建设，着力把夏南牛、悦生合、正阳花生、泌阳花菇、平舆白芝麻、正道小磨油等培育成国内知名品牌，发展一批“全链条、全循环、高质量、高效益”的农业产业化集群，力争省级农业产业化集群达到15个，农产品加工业产值达1250亿元。引导农民积极调整种植结构，促进花卉、苗木、林果、药材等特色高效农业发展。实施都市生态农业发展三年计划，重点在中心城区和确山县、汝南县、遂平县城周边，实施一批都市生态农业项目。切实加强农产品质量监管和检测体系建设，保障农产品质量安全。积极培育各类新型农业经营主体。在稳定家庭经营的基础上，支持种养大户、家庭农场、农民合作社、产业化龙头企业等新型经营主体发展，培养新型职业农民，推进多种形式的适度规模经营，力争全年新增1000家以上农民合作社，发展家庭农场500家，培育100家市级“百强示范社”，全市土地流转面积达到400万亩。加强农业基础能力建设。继续抓好骨干河道与中小河流治理、大型灌区续建配套及节水改造、小农水重点县建设等水利项目，完成石门水库除险加固任务，争取开工板桥水库及24座小型病险水库除险加固和洪汝河治理等项目建设，治理水土流失面积75平方公里。大力实施精准扶贫。健全精准扶贫工作机制，深入推进集中连片地区扶贫开发，持续打好扶贫攻坚战，实现全市贫困村定点扶贫全覆盖。切实抓好贫困乡村产业发展，着力打造“一村一品”。发挥好30个示范村的作用，扎实做好130个贫困村的整村推进工作，确保完成10.8万贫困人口脱贫任务。深入推进新农村建设。按照“五规合一”要求，以县为单位编制完善新农村建设规划，分类推进新农村综合发展、特色发展。持续推进97个已开工的新农村社区建设，引导群众尽快搬迁入住。加强农村水、电、路、气、房等基础设施建设，全面完成“十二五”农村饮水安全建设任务，新建改建农村公路1032公里。持续推进美丽乡村建设，深入开展农村垃圾、污水、秸秆焚烧集中整治活动，搞好硬化美化绿化，改善农村人居环境，把更多村庄建设成为宜居的美丽家园。

(六)大力发展现代服务业，积极培育新的经济增长点。坚持生产性服务业与生活性服务业并重，在改造提升传统服务业的同时，推动现代服务业加快发展。加快商务中心区和特色商业区建设。高标准做好“两区”规划建设，进一步加大招商力度，着力引进一批投资规模大、带动能力强、业态模式新的知名企业入驻；加快市商务中心区蓝天世贸中心、置地国际广场、高铁新城、同信广场等重点项目建设，确保尽快成规模、出形象；切实抓好驿城区特色商业区、平舆上河城、上蔡黄淮商贸城、汝南中汇国际商城等项目实施。全年完成“两区”投资80亿元以上，新开工千万元以上项目20个。加快现代物流业发展。科学编制物流业发展规划，着力推进粮食、医药、化工、冷链、快递等行业物流发展，重点抓好福汉兴公路港、中部汽贸物流园、恒兴物流园建设，积极争取火车站货场南迁，着力打造豫南区域物流中心。加快老城区传统零售业升级改造，不断提升商贸服务水平。大力实施“万村千乡”工程，加强农产品直销店、批发市场和专业市场建设，抓好农超农社对接，推动物流网点、物流配送向农村延伸，构建覆盖城乡的现代流通体系。加快文化旅游产业发展。高水平编制“十三五”旅游产业发展规划，突出抓好嵖岈山国家旅游度假区、老乐山生态文化产业园、千年岭国家农耕文化公园、鲍棚美丽乡村等项目建设，实现嵖岈山景区创5A级景区目标，全市旅游业综合收入突破120亿元。完善文化影视产业扶持政策，放宽市场准入，加快文化产业园区和示范基地建设，推动演艺、创意、网络、文物复仿等产业发展。促进房地产业平稳健康发展。加强房地产市场运行及发展趋势研判，客观引导房地产投资和消费；依法规范房地产市场，不断提高规划建设和物业管理水平；优化住房供应结构，满足多数群众的住房需求。加快电子商务产业发展。认真落实各项扶持政策，结合国家实施的“互联网+”行动计划，发挥我市电子商务产业园和跨境电子商务学院的作用，集中引进一批电子商务企业和“互联网+”应用项目。抓好阿里巴巴·驻马店产业带运营发展，鼓励我市商贸、工业企业线上交易，扶持发展一批“电商村”、“淘宝村”，努力打造我市电子商务产业发展新高地。加快健康养老产业发展。发挥政府主导作用和社会力量主体作用，坚持居家养老、机构养老和其他多种形式养老并举，促进养老服务与医疗、家政、保险、教育、健身、旅游等相关领域互动发展。规划建设市健康养老产业园；加快康复护理院等项目建设；进一步完善配套措施，促进家庭服务业发展。

(七)全面深化改革，增强经济发展的动力和活力。认真落实上级改革政策，聚焦影响经济社会发展的突出障碍和群众反映强烈的突出问题，积极推动重点领域和关键环节的改革，努力交出一份为发展加力、让人民受益的改革答卷。强化行政管理体制改革。全面完成市、县政府机构改革，完成党政机关公务用车制度改革。深入推进事业单位分类改革，开展事业单位法人治理结构试点，完成交通执法体制改革任务。继续取消和下放一批行政审批事项，全部取消非行政许可审批，建立规范行政审批的管理制度。深化商事制度改革，进一步简化注册资本登记，逐步实现“三证合一”，清理规范中介服务。加快财税体制改革。认真落实新《预算法》，深化预算管理制度改革，实行全面规范、公开透明的预算管理制度，除法定涉密信息外，所有部门预算都要公开。把政府性债务分类纳入全口径预算管理，实行统一归口管理和规模控制。进一步深化公务卡、国库集中收付制度改革，推进“营改增”、消费税、资源税等税制改革。加强预算绩效管理，严格控制“三公经费”等一般性支出，提高资金使用效率。深化金融改革。加强与兴业银行、交通银行及区域性商业银行的对接，争取引进一家股份制银行设立分支机构。依托驿城区农联社筹建驻马店农商行，完成西平县、汝南县农商行组建工作；加快汝南县、确山县村镇银行组建步伐，力争实现县域全覆盖。加强上市企业培育，力争枫华种业、全宇生物、龙凤山牧业、中多铝业等企业在“新三板”挂牌上市。不断创新金融产品，简化审批程序，争取信贷规模，加强银企合作，加大对实体经济的支持力度，力争全市各项贷款余额突破1000亿元，存贷比突破50%。不断扩大保险覆盖领域，积极发展环境污染、医疗责任保险和涉农保险，利用好保险资金、社保基金的投资功能。积极推进投融资改革。大幅缩减政府核准投资项目范围和前置审批，下放项目核准权限，实行项目核准网上并联办理，将126项工商登记改为后置审批。探索设立新型城镇化发展子基金，争取省里资金支持；积极推行政府与社会资本合作模式，引导社会资本投入城市基础设施、公用事业等重点项目建设。深化医药卫生体制改革。统筹推进医疗保障、医疗服务、公共卫生、药品供应和监管体制改革，全面深化县级公立医院综合改革，鼓励发展社会办医。完善全民医保体系、重特大疾病保障和乡、村两级基本药物制度，推进基本公共卫生服务均等化。全面深化农村改革。深入推进农村集体产权制度改革，积极稳妥开展土地承包经营权确权登记颁证工作，西平、确山完成整县推进，其他县区完成50%。稳步推进农村土地制度改革，加快房地一体的农村宅基地使用权确权颁证，建立农村产权交易市场。深化集体林权制度、农电体制改革。加快农村金融改革，创新金融服务产品，探索农村产权抵押贷款办法，为农业农村发展提供金融服务。加快推进国有企业改革。市直部门所属企业全面完成脱钩划转，按照分类指导、一企一策的原则，积极探索“壳公司”改革方式，稳步推进市属32家国有商贸流通企业改革。加强国有资产监管，防止国有资产流失。同时，继续深化社会事业、住房公积金等改革。(八)持续改善生态环境，推进美丽驻马店建设。把生态文明建设融入经济社会发展全过程，大力实施生态建设提升工程，努力打造美丽驻马店。持续加强生态保护。认真贯彻新环保法，落实属地管理责任，完善生态补偿机制，坚持源头严防、过程严管、后果严惩，切实保护好我们的青山绿水。加强林业生态建设，抓好天然林保护、退耕还林、城郊防护林等项目建设，完成造林20.8万亩、森林抚育和改造12.5万亩。加大水土保持执法力度，严厉打击各种违法行为。推进生态系统建设修复，完成板桥水库、宿鸭湖水库流域生态修复实施计划编制工作。实行最严格的水资源管理制度，加强饮用水源地保护，推进节水型社会建设；巩固“三乱两超”整治成果，做好西部山区和淮河流域生态保护工作，依法严厉打击滥采乱挖行为。加快练江河、小清河水系治理，推进金顶湖、玉皇湖水库项目前期工作，实施引水入城工程，着力打造水生态示范市。深入开展污染防治。持续实施“蓝天工程”、“碧水工程”、“绿地工程”和“乡村清洁工程”，全面启动生态市和中心城区环保模范城创建工作。认真落实大气污染防治措施，积极推进脱硫脱硝除尘、黄标车和老旧车淘汰及汽车尾气、城市施工、道路扬尘治理，深入开展中心城区燃煤锅炉、商业门店燃煤灶具拆改集中行动。加强水污染防治，重点抓好练江河、黑河等河流综合整治及水污染源、农业面源污染治理，第三污水处理厂、中水厂建成投用，开工建设禇堂生活垃圾分类处理项目，启动第四污水处理厂前期工作。扎实推进节能减排。推行合同能源管理，控制能源消费总量，加强工业、交通、建筑等重点领域节能。实施节能环保技术装备产业化示范工程，支持企业实施节能环保技改及新上项目采用先进工艺技术。切实做好总量减排，强化重点减排工程建设，加强减排项目运行监管，确保完成减排目标任务。构建生态文明建设长效机制。完善能源消费总量和重点污染物排放总量预算管理制度，建立健全生态环境补偿和环境损害鉴定评估机制，实施排污权、碳排放、林权交易制度，开展环境污染第三方治理试点，探索把环境质量反降级作为刚性约束条件，确保绿色发展者受益，使损害环境者付出代价，让驻马店的天更蓝、水更清、地更绿，让人民群众呼吸上新鲜空气、喝上放心水。

(九)切实保障改善民生，努力保持社会和谐稳定。切实加大民生投入力度，继续实施好十大民生工程和中心城区六件惠民实事，使发展成果更多惠及人民群众。加强就业和社会保障。实施好高校毕业生就业促进计划和创业引领计划，鼓励到基层就业、到民营企业就业，围绕现代农业、新兴产业创业。深入实施全民技能振兴工程和农民工职业技能提升计划，统筹抓好农村转移劳动力、城镇困难人员、退役军人就业工作，完成各类职业技能培训16万人次，城镇新增就业4.8万人，新增农村劳动力转移就业7万人。提高城镇中低收入居民收入，完善最低工资标准调整机制，落实机关事业单位养老保险制度改革措施，同步完善工资制度，在县以下机关建立公务员职务和职级并行制度。依法治理拖欠农民工工资问题，切实保障农民工合法权益。继续扩大社会保险覆盖范围，提高城乡居民基础养老金标准，企业退休人员基本养老金再提高10%。继续提高城乡低保、农村五保和优抚对象补助标准。全面实施临时救助制度，让遇到急难特困群众求助有门、受助及时。加大保障性安居工程建设力度，建设保障性住房3.1万套。办好人民满意的教育。认真编制实施“两个规划”、“两个计划”，新建、改扩建幼儿园122所，促进学前教育普惠发展；实行城乡统一的义务教育学校公用经费，扩充城镇义务教育资源，改善贫困地区农村义务教育办学条件，新建、改扩建一批标准化学校；推进特殊教育学校标准化建设，提升特殊教育办学水平。推动高中教育优质特色发展。落实农民工随迁子女在流入地接受义务教育政策。实施职业教育二期攻坚计划，以黄淮学院为龙头，以驻马店职业技术学院和各类职业中专为支撑，逐步建立完善的现代职业教育体系。落实普惠性中等职业教育政策，从秋季起对全日制中职在校生全免学费。加快推进市职教园区建设，完成投资13亿元以上，实现农校、卫校搬迁入驻，做好驻马店技师学院和驻马店幼儿师专申办工作。支持黄淮学院转型发展，办好应用技术大学。提升群众健康水平。全面完成医疗卫生事业三年九大攻坚行动计划，继续抓好县级医院和乡镇卫生院改扩建等项目建设。加强重大疾病防控。完善城乡居民基本医保，财政补助标准每人每年再提高60元，基本公共卫生服务经费财政补助标准从人均35元提高到40元。完善新农合大病保险，全面推行城镇居民大病保险，实施疾病应急救助制度。加强计划生育服务管理，实现免费孕前优生健康检查城乡全覆盖。加快妇女儿童医院建设，市第二中医院建成投用，市中心医院北区分院开工建设。加快养老服务体系建设，市社会福利院和各县福利中心全部建成投用。大力发展各项社会事业。加快交通事业发展，积极推进107国道升级改造项目建设，加大S333汝南至驿城区段一级公路改建项目争取力度，强力推进S219永定线正阳至大林等7条干线公路建设，京港澳高速驻马店段改扩建工程建成通车。繁荣发展文学艺术、新闻出版、广播影视等事业，重视文物、非物质文化遗产保护。积极筹建市气象科普馆，加快推进驻马店日报报业大厦、妇女儿童活动中心项目建设，启动市科技馆、艺术中心和青少年活动中心前期工作。继续抓好“三馆一站”和市老年健身活动中心免费开放、“舞台艺术送农民”等文化惠民实事。加强公共体育设施建设，开展全民健身运动，筹办组织好市第二届运动会暨首届全民健身大会。加强残疾人就业培训，做好0～6岁残疾儿童抢救性康复工作。完善扶持政策，推动慈善事业健康发展。加强和创新社会治理。牢固树立“大平安”理念，着眼“双安”、推进“双治”、强化“双基”，健全“四项基础制度”。全面推行重大决策社会稳定风险评估机制，建立县、乡、村三级矛盾纠纷调处化解体系，抓好阳光信访、逐级走访、诉访分离、积案化解等工作，有效预防和化解社会矛盾。健全隐患排查治理和安全预防控制体系，强化食品药品监管和质量技术监督，坚决遏制重特大事故发生。规范市场秩序，打击侵权假冒行为。加强应急管理，提高公共突发事件防范处置和防灾、救灾、减灾能力。创新立体化社会治安防控体系，构建“一村一警”长效机制，依法严厉打击暴力犯罪和黑恶势力，做好反恐工作，确保社会和谐稳定、人民安居乐业。着力防控各类风险。随着经济增速的放缓，各种潜在矛盾将日益凸显，我们务必高度重视，未雨绸缪、主动应对。及早安排并妥善化解部分行业特困企业和金融、房地产、地方债务、社会领域积累的风险，坚决守住不发生区域性、系统性风险底线。特别是面对非法集资高发态势，要加强宣传引导，使全社会认清其严重危害、远离非法集资；对于已经查处的案件，尽快查封、变现资产，最大限度地减少群众损失，最大限度地减少社会震动；对于新发现的案件，保持高压态势，发现一起查处一起，让非法牟利者付出应有的代价。金庸小说

加强国防教育和国防后备力量建设，支持驻驻解放军、武警部队、预备役部队、民兵和人民防空建设，深入开展双拥共建，巩固军政军民团结，促进军民融合深度发展，实现第四届“双拥模范城”创建目标。支持工会、共青团、妇联等人民团体发挥桥梁纽带作用，注重发挥工商联等社团作用。认真做好统计、民族宗教、外事侨务、档案、史志、气象、对口援疆等工作。

科学编制“十三五”规划。认真开展调研，广泛征求意见，切实加强对接，提高前瞻性、战略性，深入谋划重大建设项目、重大政策措施，争取一批重大项目和事项列入国家、省里规划。

三、切实加强政府自身建设

适应新形势、新任务，坚持以建设法治政府、服务政府、高效政府、创新政府、廉洁政府为重点，切实加强政府自身建设，不断提升政府治理水平。

(一)坚持依法行政。认真落实全面推进依法治国战略，坚持尊法、学法、守法、用法，忠实履行宪法和法律赋予的职责，所有行政行为都要于法有据，任何政府部门都不得法外设权，切实做到法无授权不可为、法定职责必须为。深化行政执法体制改革，严格规范公正文明执法，加快推进综合执法，全面落实行政执法责任制，一切违法违规行为都要追究，一切执法不严不公现象都必须纠正。加强法治宣传教育，完成“六五”普法任务。严格依法行政考核，把政府工作全面纳入法治轨道。

(二)转变政府职能。正确处理好政府和市场的关系，用好有形的手和无形的手，充分发挥好市场在资源配置中的决定性作用。加快推进政企分开、政事分开、政资分开、政府与中介组织分开，强化公共服务、市场监管、社会治理、环境保护等职能，提供基本公共服务尽可能采取购买服务方式，第三方提供的事务性管理交给市场或社会办理。进一步简政放权，严格执行行政审批目录清单，减少行政审批事项。制定市场准入负面清单，公布市政府部门权力清单、责任清单。深化政务公开，依法及时、准确地向社会公开政府信息，确保权力公开透明、规范运行。

(三)提升行政效能。积极推进行政审批便利化改革，认真落实联审联批、限时办结制、模拟审批、全程代理和网上办理等制度，缩短审批时间，提高办事效率。把抓落实摆到更加突出位置，对市委、市政府部署的重点工作，特别是在招商引资、项目建设、城市发展、民生改善等大事要事上，保持定力、持续推进、一抓到底，以敢于担当的锐气、抓铁有痕的作风和钉钉子精神，加压使劲、久久为功，善做善成。加大督察力度，强化行政问责，坚决纠正不作为、乱作为，坚决克服懒政怠政，坚决惩处失职渎职，真正形成倒逼机制，确保各项工作落到实处。

(四)着力创新发展。面对繁重的发展任务和经济发展新常态，广大公务员特别是各级领导干部要加强学习，准确把握发展规律，努力摆脱“换挡焦虑”、克服“本领恐慌”，研究新方法，用好新机遇，学会用改革办法、创新手段推动发展。进一步解放思想，善于破除僵化的思维模式，树立法治思维法治方式、市场思维市场方式，以新思维研究新问题，以新思路落实新任务，以新举措开创加快发展的新局面。强化问题导向，对影响工作推进的关键问题、制约长远发展的重大难题、群众反映强烈的热点问题，坚持用改革创新的办法，加强调查研究，着力进行破解，努力扫除障碍，加快发展步伐。

(五)加强廉政建设。扎实开展“三严三实”专题教育，认真遵守政治纪律和政治规矩，认真落实中央八项规定精神，坚持不懈纠正“四风”，严格执行国务院“约法三章”，坚决不搞劳民伤财的形象工程、政绩工程。认真落实党风廉政建设主体责任，强化“一岗双责”，把廉洁从政贯彻到政府工作各个环节。完善财政资金支出、工程项目招投标、国有企业改革及公共资源交易领域各项制度，以权力“瘦身”为廉政“强身”，紧紧扎住制度围栏，坚决打掉寻租空间，努力铲除腐败土壤。强化权力运行制约监督，加强行政监察和审计监督，强化廉政教育，遵守廉政准则，保持清廉本色，以廉政建设和反腐败斗争实效保障发展、取信于民!

各位代表!作为人民政府，我们一定严格执行市人大及其常委会的决议决定，政府重大决策及时向人大及其常委会报告，自觉接受法律和工作监督，主动接受市政协的民主监督，虚心接受人民群众社会监督和媒体舆论监督，认真办理人大代表建议和政协提案，广泛听取各民主党派、工商联、无党派人士、人民团体的意见，让人民更好地监督政府，让政府工作更好地体现人民意愿，决不允许慵懒散奢，决不允许弄权渎职，决不允许损害群众利益!

各位代表!新常态赋予新使命，蕴含新机遇，呼唤新作为。推动经济持续健康发展，不断增进民生福祉，实现社会和谐安定,需要众志成城、合力向前。让我们在省委、省政府和市委的正确领导下，进一步解放思想、开拓创新、扎实工作，努力创造经得起实践和历史检验的新业绩，为建设富强驻马店、文明驻马店、平安驻马店、美丽驻马店不断开创富民强市加快崛起的新局面而努力奋斗!
